# Comparison of the Antimicrobial Efficacy of Sodium Hypochlorite, Silver Nanoparticles, Chitosan-Capped Silver Nanoparticles, and Chlorhexidine-Loaded Silver Nanoparticles as Endodontic Irrigants Against Enterococcus faecalis: An In Vitro Study

**DOI:** 10.7759/cureus.113902

**Published:** 2026-08-03

**Authors:** Athulya B Mohan, Aiswarya Jayaram, Anju Prakash, Sabir Muliyar, Vivek VJ, Suraj U

**Affiliations:** 1 Conservative Dentistry and Endodontics, Malabar Dental College and Research Center, Malappuram, IND

**Keywords:** chitosan-capped silver nanoparticles, chlorhexidine-loaded silver nanoparticles, endodontic irrigants, enterococcus faecalis, silver nanoparticles, sodium hypochlorite

## Abstract

Introduction

Persistent endodontic infections are frequently associated with *Enterococcus faecalis*, a microorganism capable of surviving harsh intracanal conditions and penetrating dentinal tubules. Although sodium hypochlorite remains the gold-standard irrigant, nanoparticle-based irrigants have emerged as promising alternatives due to their enhanced antimicrobial properties.

Aim

The aim of the study is to compare and evaluate the antimicrobial efficacy of sodium hypochlorite, silver nanoparticles, chitosan-capped silver nanoparticles, and chlorhexidine-loaded silver nanoparticles against *E. faecalis* in an in vitro model.

Materials and methods

Forty-five extracted mandibular premolars were divided into five groups (n = 9). Following standardization of root length and biomechanical preparation, canals were inoculated with *E. faecalis* (ATCC 29212) and incubated for 14 days. The specimens were irrigated with the respective test solutions and subjected to passive ultrasonic agitation. Microbial samples were collected using sterile paper points, and colony-forming units (CFU) were determined. Data were analyzed using one-way analysis of variance (ANOVA) and Tukey’s post hoc test with significance set at p < 0.05.

Results

A statistically significant difference was observed among the groups (p < 0.001). Silver nanoparticles demonstrated the greatest antimicrobial efficacy, followed by sodium hypochlorite, chitosan-capped silver nanoparticles, and chlorhexidine-loaded silver nanoparticles. The control group exhibited the highest bacterial counts.

Conclusion

Silver nanoparticles exhibited superior antimicrobial activity against *E. faecalis* compared with the other irrigants tested and may represent a promising alternative intracanal irrigant. This study highlights the potential of nanoparticle-based irrigants as a future alternative to conventional endodontic irrigation protocols.

## Introduction

Microorganisms are the primary etiological factor responsible for pulpal and periapical diseases, and their complete elimination remains the primary objective of endodontic treatment [1]. Despite significant advancements in instrumentation techniques and irrigation protocols, complete disinfection of the root canal system remains challenging because of the anatomical complexity of root canals, including isthmuses, fins, lateral canals, and dentinal tubules [2,3]. These anatomical irregularities can harbor microorganisms beyond the reach of mechanical instrumentation, making irrigation an indispensable component of root canal therapy [4].

Among the microorganisms associated with persistent endodontic infections, *Enterococcus faecalis* is regarded as one of the most resistant and clinically significant species [5]. This facultative anaerobic Gram-positive coccus is frequently isolated from previously treated root canals exhibiting post-treatment apical periodontitis [6-8].

Root canal irrigation plays a pivotal role in reducing microbial load and removing organic debris from inaccessible regions of the canal system [9-11]. Sodium hypochlorite (NaOCl) remains the most commonly used irrigant owing to its antimicrobial properties and tissue-dissolving capability. However, concerns regarding cytotoxicity and limited residual antimicrobial activity have prompted the search for alternative irrigants [12,13].

Recent advances in nanotechnology have introduced silver nanoparticles (AgNPs), chitosan-capped AgNPs (CS-AgNPs), and chlorhexidine-loaded AgNPs (CHX-AgNPs) as potential endodontic irrigants. These materials possess unique physicochemical characteristics that may enhance antimicrobial activity and biofilm disruption [14]. Therefore, the present study aims to compare the antimicrobial efficacy of NaOCl, AgNPs, CS-AgNPs, and CHX-AgNPs against *E. faecalis* in an in vitro root canal infection model.

## Materials and methods

Sample size calculation and teeth selection

The sample size was calculated using G*Power software (version 3.1; Heinrich-Heine-Universität Düsseldorf, Düsseldorf, Germany) for a one-way analysis of variance (ANOVA) with five independent groups. Assuming a large effect size (Cohen's f = 0.55), a two-sided significance level (α) of 0.05, and a statistical power of 80% (1 − β = 0.80), the minimum required sample size was 45 specimens.

Forty-five extracted human mandibular premolars with single, straight root canals and mature apices were selected for this study. Mandibular premolars were chosen because their relatively uniform root canal anatomy minimizes anatomical variability and allows standardization of canal preparation and irrigation procedures. Furthermore, the presence of oval or irregular canal cross-sections may limit complete debridement by mechanical instrumentation alone, thereby warranting meticulous chemo-mechanical preparation to ensure effective disinfection. These teeth were also readily available following orthodontic extractions, ensuring uniform sample selection and enhancing the reproducibility of the study. Teeth exhibiting fractures, cracks, root caries, root resorption, or canal obliteration were excluded.

Specimen preparation

The teeth were decoronated and root lengths standardized to 14 mm. Root canal instrumentation was performed using HyFlex CM rotary instruments (Coltene Holding AG, Altstätten, Switzerland) up to size 40/0.04 taper. Each instrumented canal was irrigated with 5 mL of 5.25% NaOCl. Final irrigation was carried out using 2 mL of 17% ethylenediaminetetraacetic acid (EDTA) followed by saline. The root apex was restored with light-cured composite resin (3M, Saint Paul, MN, USA), and the external root surface was covered with two layers of nail varnish. Samples were then sterilized at 121°C for 15 minutes at 15 psi pressure in an autoclave.

Microbial contamination

**Figure 1:**
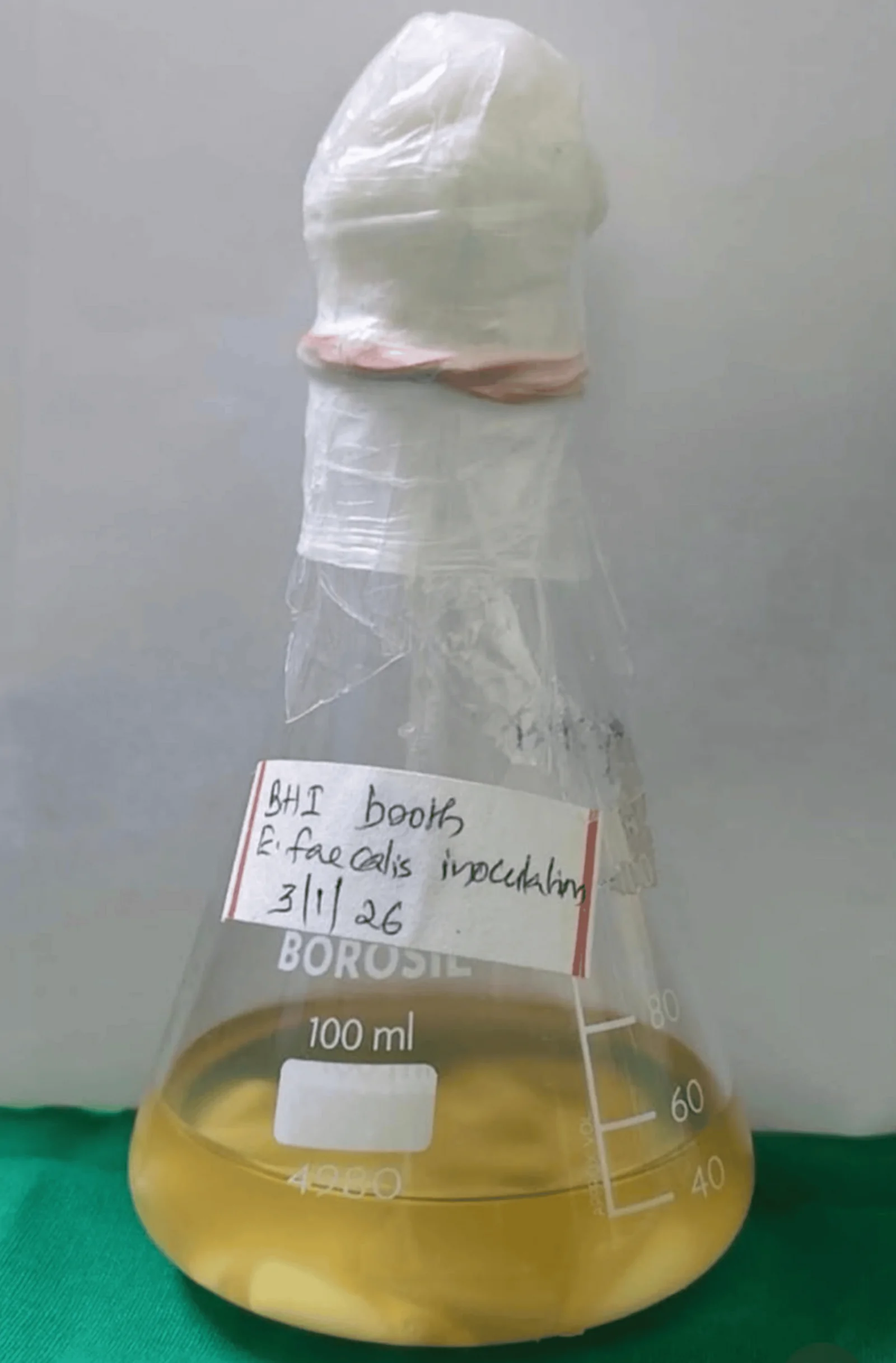
Brain-heart infusion (BHI) broth: Enterococcus faecalis inoculation.

*E. faecalis* (ATCC 29212) was cultured in brain-heart infusion broth (Figure 1) and inoculated into the prepared canals.

Test irrigants

Irrigants used in the study were 5.25% NaOCl; AgNPs (10-20 nm; 100 mg/mL), which were procured from 3K Nano Pvt. Ltd., New Delhi, India; CS-AgNPs (10-20 nm; 100 mg/mL) from Silica Nano Solutions, Fatehpur, Uttar Pradesh, India; and CHX-AgNPs (20-30 nm; 100 mg/mL) from 3K Nano Pvt. Ltd. The nanoparticle formulations were supplied as ready-to-use suspensions. All irrigants were stored according to the manufacturers’ recommendations and were used within their stated shelf life (one year from the date of manufacture) throughout the study.

**Figure 2:**
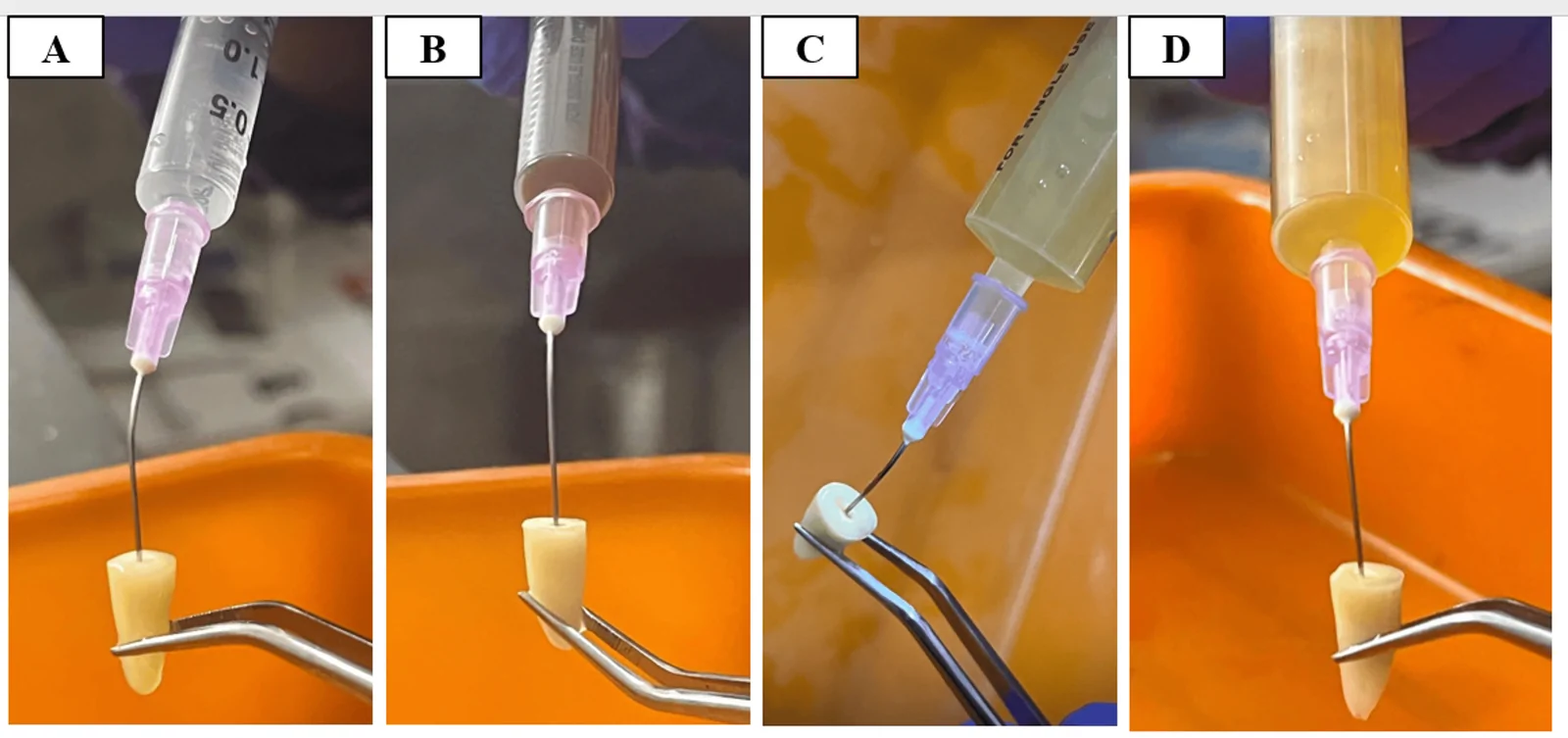
Irrigation of tooth samples with different irrigating solutions. (A) Sodium hypochlorite; (B) silver nanoparticles; (C) chitosan-capped silver nanoparticles; (D) chlorhexidine-loaded silver nanoparticles.

Samples were incubated at 37°C for 14 days with replenishment of broth every 48 hours. The teeth were then randomly allocated into five experimental groups (n = 9): Group I: NaOCl, Group II: AgNPs, Group III: CS-AgNPs, Group IV: CHX-AgNPs, and Group V: control group. In Groups I-IV, canals were irrigated with 5 mL of the respective test solutions for one minute and subjected to passive ultrasonic irrigation using the Eighteeth Medical Ultra X Cordless Ultrasonic Activator (Changzhou Sifary Medical Technology Co., Ltd., Changzhou, China) and finally flushed with 5 mL sterile saline. Group V served as the untreated control and received neither irrigation nor passive ultrasonic activation (Figure 2).

Microbiological analysis

**Figure 3:**
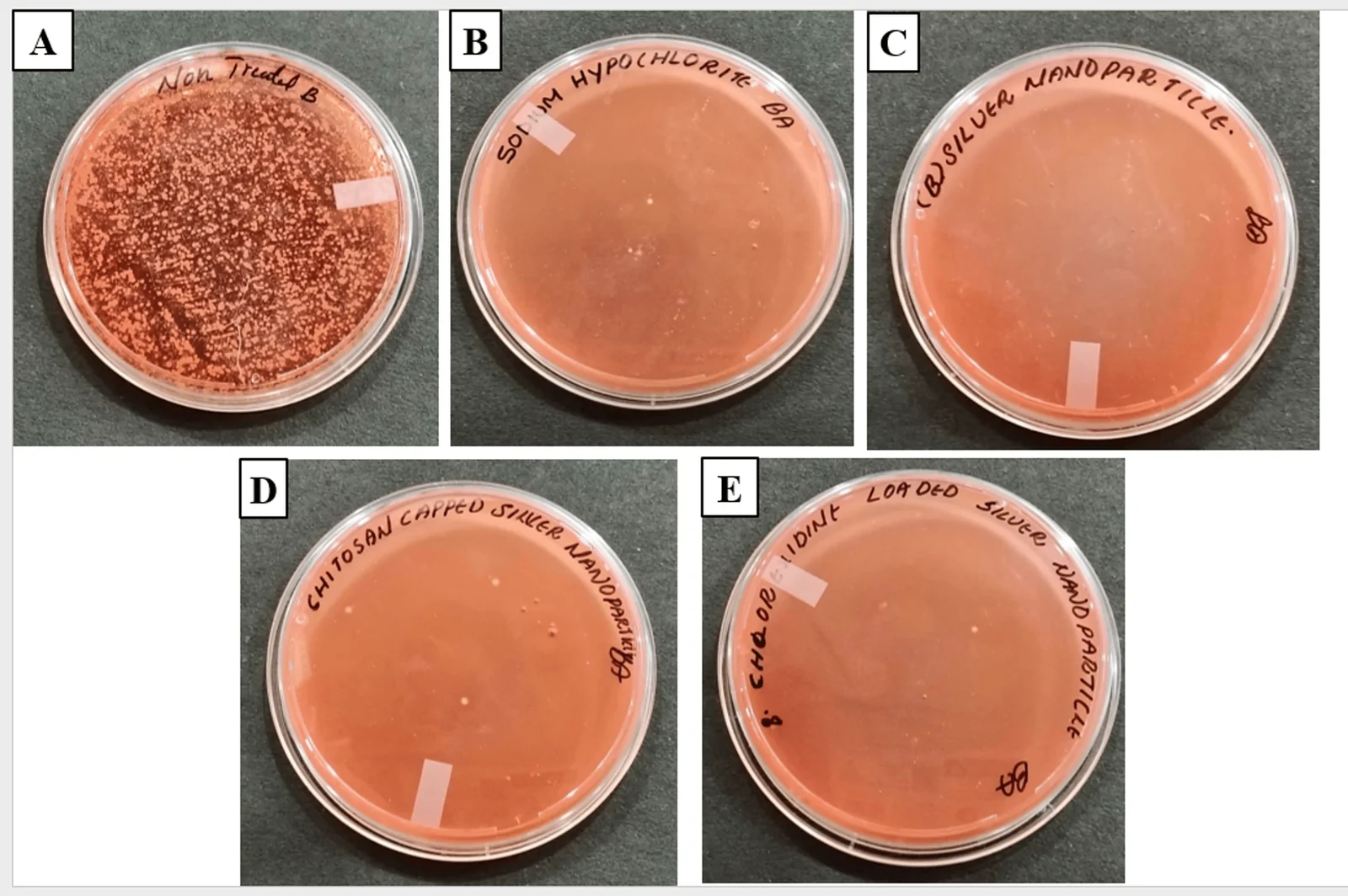
Agar plates showing antibacterial activity against Enterococcus faecalis. (A) Non-treated control showing abundant bacterial growth. (B) Sodium hypochlorite showing minimal bacterial growth. (C) Silver nanoparticles showing marked reduction in bacterial colonies. (D) Chitosan-capped silver nanoparticles showing minimal bacterial growth. (E) Chlorhexidine-loaded silver nanoparticles showing minimal bacterial growth.

Post-irrigation microbial samples were collected using sterile paper points. Samples were serially diluted, plated on culture media, and incubated, and colony-forming units (CFU) were determined by counting (Figure 3).

Statistical analysis

Data were analyzed using descriptive statistics, one-way ANOVA, and Tukey’s honestly significant difference (HSD) post hoc test. Statistical significance was set at p < 0.05.

## Results

The mean log₁₀ CFU counts of *E. faecalis* following irrigation with the tested solutions are presented in Table 1.

**Table 1:** Mean ± SD of log₁₀ values with ANOVA. One-way ANOVA test: p < 0.05 is statistically significant. ANOVA: analysis of variance; NaOCl: sodium hypochlorite; AgNP: silver nanoparticle; CHX: chlorhexidine

	Material	n	Mean log₁₀ ± SD	F-value	p-value
Group 1	NaOCl	9	3.301 ± 0.010	96.52	<0.001
Group 2	AgNP	9	0.000 ± 0.000		
Group 3	Chitosan	9	3.477 ± 0.021		
Group 4	CHX-loaded	9	3.477 ± 0.021		
Group 5	Control	9	5.665 ± 0.015		

The control group exhibited the highest bacterial count (5.665 ± 0.015), whereas the AgNP group demonstrated complete inhibition of bacterial growth with a mean log₁₀ value of 0.000 ± 0.000. The NaOCl group showed a mean log₁₀ value of 3.301 ± 0.010, while both CS-AgNP and CHX-AgNP groups recorded mean values of 3.477 ± 0.021.

One-way ANOVA revealed a highly significant difference among the five groups (F = 96.52, p < 0.001), indicating that the antimicrobial efficacy varied significantly between the irrigants tested. Tukey’s HSD post hoc test was applied for pairwise comparisons between the groups. A p-value of less than 0.05 was considered statistically significant for all analyses and is shown in Table 2.

**Table 2:** Tukey HSD post hoc test for pairwise comparison of log₁₀ values. Tukey HSD test: p < 0.05 is statistically significant. HSD: honestly significant difference; NaOCl: sodium hypochlorite; AgNP: silver nanoparticle; CHX: chlorhexidine

Comparison	Mean difference	p-value	Significance
NaOCl vs AgNP	3.301	<0.001	Significant
NaOCl vs chitosan	-0.176	0.021	Significant
NaOCl vs CHX-loaded	-0.176	0.021	Significant
NaOCl vs control	-2.364	<0.001	Significant
AgNP vs chitosan	-3.477	<0.001	Significant
AgNP vs CHX-loaded	-3.477	<0.001	Significant
AgNP vs control	-5.665	<0.001	Significant
Chitosan vs CHX-loaded	0.000	1.000	Not significant
Chitosan vs control	-2.188	<0.001	Significant
CHX-loaded vs control	-2.188	<0.001	Significant

Post hoc pairwise comparisons using Tukey’s HSD test demonstrated statistically significant differences between most groups. AgNPs showed significantly greater antimicrobial efficacy than NaOCl, CS-AgNPs, CHX-AgNPs, and the control group (p < 0.001). NaOCl also differed significantly from both CS-AgNPs and CHX-AgNPs (p = 0.021). However, no statistically significant difference was observed between the CS-AgNP and CHX-AgNP groups (p = 1.000), suggesting comparable antimicrobial performance. Overall, the antimicrobial efficacy of the tested irrigants against E. faecalis was ranked as AgNP > NaOCl > CS-AgNP ≈ CHX-AgNP > control.

## Discussion

Successful endodontic treatment depends on effective elimination of microorganisms from the root canal system [14,15]. Among the microorganisms implicated in persistent endodontic infections, *E. faecalis* is considered one of the most resistant species because of its ability to penetrate dentinal tubules, survive under nutrient-deprived conditions, form biofilms, and withstand intracanal medicaments [7-16].

Although NaOCl remains the gold-standard irrigant because of its excellent tissue-dissolving ability and broad-spectrum antimicrobial activity, it has several well-recognized disadvantages, including cytotoxicity when extruded beyond the apex, unpleasant taste and odor, reduction in dentin flexural strength with prolonged exposure, and inability to provide prolonged antibacterial substantivity [17]. These shortcomings have encouraged the investigation of nanoparticle-based alternatives that combine potent antimicrobial effects with improved biocompatibility.

Nanoparticles, owing to their extremely small particle size (1-100 nm), possess unique physicochemical characteristics such as a high surface area-to-volume ratio, increased surface reactivity, enhanced diffusibility, and improved penetration into inaccessible areas of the root canal system. These properties allow nanoparticles to interact more efficiently with bacterial biofilms and microorganisms embedded within dentinal tubules.

The results demonstrated statistically significant differences among all groups (p < 0.001), compared to the control, indicating that the tested irrigants exhibited varying antimicrobial effects against *E. faecalis*. AgNPs showed the highest antimicrobial efficacy with complete inhibition of bacterial growth, whereas the control group exhibited the highest bacterial counts.

NaOCl demonstrated the second-highest antimicrobial efficacy. The antimicrobial effect of NaOCl is primarily attributed to the formation of hypochlorous acid and chloramine compounds, which oxidize essential bacterial enzymes and disrupt cellular metabolism [18,19]. Despite its effectiveness, NaOCl did not achieve complete bacterial elimination in the present study, which may be due to the ability of *E. faecalis* to penetrate deeply into dentinal tubules and survive in protected anatomical niches.

The superior antimicrobial activity observed with AgNPs may be attributed to their unique physicochemical properties [20]. Due to their nanoscale dimensions, AgNPs possess a large surface area-to-volume ratio, enabling intimate interaction with bacterial cell membranes. Silver ions released from nanoparticles can disrupt membrane permeability, generate reactive oxygen species, interfere with DNA replication, inhibit essential enzymes, and ultimately lead to bacterial cell death [21,22]. Additionally, their small particle size (1-100 nm) facilitates penetration into dentinal tubules and biofilm matrices, allowing them to reach microorganisms inaccessible to conventional irrigants [23]. These mechanisms collectively explain the complete suppression of *E. faecalis* observed in the present study.

CS-AgNPs exhibited lower antimicrobial efficacy than both AgNPs and NaOCl. Chitosan is a natural polysaccharide with intrinsic antimicrobial properties resulting from electrostatic interactions between its positively charged amino groups and negatively charged bacterial cell walls [24]. These interactions increase membrane permeability and promote leakage of intracellular contents [25,26]. Furthermore, chitosan possesses chelating properties that may contribute to biofilm disruption. Although chitosan enhances nanoparticle stability and biocompatibility, the present findings suggest that capping AgNPs with chitosan may reduce the availability of free silver ions, thereby limiting their antimicrobial potential compared with uncapped AgNPs [27].

Similarly, CHX-AgNPs demonstrated antimicrobial activity but did not outperform AgNPs or NaOCl. Chlorhexidine is well known for its substantivity and broad-spectrum antimicrobial effect [28]. The incorporation of chlorhexidine into AgNPs theoretically combines the substantivity of chlorhexidine with the bactericidal action of silver [29,30]. However, the present study found no statistically significant difference between CHX-AgNPs and CS-AgNPs. This observation suggests that under the experimental conditions employed, the anticipated synergistic effect may not have translated into superior antibacterial performance.

The absence of a significant difference between the CS-AgNP and CHX-AgNP groups indicates that both formulations exhibited comparable efficacy against *E. faecalis*. Similar findings have been reported in nanoparticle-based antimicrobial studies where modifications intended to enhance nanoparticle activity did not necessarily result in significantly improved antibacterial outcomes.

A notable finding of the present investigation was the complete suppression of bacterial growth in the AgNP group. This observation highlights the potential of nanoparticle-based irrigants as future alternatives to conventional endodontic irrigation protocols. However, further in vivo studies and randomized clinical trials are required to establish their safety, efficacy, and long-term clinical performance before routine clinical application.

The present study has certain limitations. As an in vitro investigation, it cannot fully reproduce the complex biological conditions of the clinical root canal system. The study employed a mono-species biofilm model, whereas endodontic infections are polymicrobial in nature. Additionally, only antimicrobial efficacy was evaluated, and other important properties such as tissue dissolution, smear layer removal, and biocompatibility were not assessed. Interaction among the tested irrigants was not assessed. Therefore, further in vivo studies and randomized clinical trials are required to validate these findings.

## Conclusions

Within the limitations of this in vitro study, all tested irrigants demonstrated antimicrobial activity against *E. faecalis*. AgNPs exhibited the highest antimicrobial efficacy and achieved complete bacterial inhibition. NaOCl demonstrated significantly greater antimicrobial activity than CS-AgNPs and CHX-AgNPs. No significant difference was observed between CS-AgNPs and CHX-AgNPs. However, further in vivo studies and randomized clinical trials are required to establish their safety, efficacy, and long-term clinical performance before routine clinical application.

## References

[1] Wong J, Manoil D, Näsman P, Belibasakis GN, Neelakantan P (2021). Microbiological aspects of root canal infections and disinfection strategies: an update review on the current knowledge and challenges. Front Oral Health.

[2] Mamat R, Nik Abdul Ghani NR (2023). The complexity of the root canal anatomy and its influence on root canal debridement in the apical region: a review. Cureus.

[3] Gomes BP, Aveiro E, Kishen A (2023). Irrigants and irrigation activation systems in endodontics. Braz Dent J.

[4] Siqueira Junior JF, Rôças ID, Marceliano-Alves MF, Pérez AR, Ricucci D (2018). Unprepared root canal surface areas: causes, clinical implications, and therapeutic strategies. Braz Oral Res.

[5] Alghamdi F, Shakir M (2020). The influence of Enterococcus faecalis as a dental root canal pathogen on endodontic treatment: a systematic review. Cureus.

[6] Rôças IN, Siqueira JF Jr, Santos KR (2004). Association of Enterococcus faecalis with different forms of periradicular diseases. J Endod.

[7] Sharma J, Jhamb S, Mehta M, Bhushan J, Bhardwaj SB, Kaur A (2025). Characterization of Enterococcus faecalis associated with root canal failures: virulence and resistance profile. J Conserv Dent Endod.

[8] Love RM (2001). Enterococcus faecalis--a mechanism for its role in endodontic failure. Int Endod J.

[9] Kandaswamy D, Venkateshbabu N (2010). Root canal irrigants. J Conserv Dent.

[10] Zehnder M (2006). Root canal irrigants. J Endod.

[11] Haapasalo M, Shen Y, Wang Z, Gao Y (2014). Irrigation in endodontics. Br Dent J.

[12] Mohammadi Z (2008). Sodium hypochlorite in endodontics: an update review. Int Dent J.

[13] Pai AR (2023). Sodium hypochlorite irrigation and safety. Br Dent J.

[14] Narayanan LL, Vaishnavi C (2010). Endodontic microbiology. J Conserv Dent.

[15] Stuart CH, Schwartz SA, Beeson TJ, Owatz CB (2006). Enterococcus faecalis: its role in root canal treatment failure and current concepts in retreatment. J Endod.

[16] Portenier I, Waltimo TM, Haapasalo M (2004). Enterococcus faecalis-the root canal survivor and ‘star’ in post-treatment disease. Endod Topics.

[17] Cai C, Chen X, Li Y, Jiang Q (2023). Advances in the role of sodium hypochlorite irrigant in chemical preparation of root canal treatment. Biomed Res Int.

[18] Pascon FM, Kantovitz KR, Sacramento PA, Nobre-dos-Santos M, Puppin-Rontani RM (2009). Effect of sodium hypochlorite on dentine mechanical properties. A review. J Dent.

[19] Siqueira JF Jr, Machado AG, Silveira RM, Lopes HP, de Uzeda M (1997). Evaluation of the effectiveness of sodium hypochlorite used with three irrigation methods in the elimination of Enterococcus faecalis from the root canal, in vitro. Int Endod J.

[20] Qamer S, Romli MH, Che-Hamzah F, Misni N, Joseph NM, Al-Haj NA, Amin-Nordin S (2021). Systematic review on biosynthesis of silver nanoparticles and antibacterial activities: application and theoretical perspectives. Molecules.

[21] Afkhami F, Forghan P, Gutmann JL, Kishen A (2023). Silver nanoparticles and their therapeutic applications in endodontics: a narrative review. Pharmaceutics.

[22] Yin IX, Zhang J, Zhao IS, Mei ML, Li Q, Chu CH (2020). The antibacterial mechanism of silver nanoparticles and its application in dentistry. Int J Nanomedicine.

[23] Bhandi S, Mehta D, Mashyakhy M (2021). Antimicrobial efficacy of silver nanoparticles as root canal irrigant's: a systematic review. J Clin Med.

[24] Wei D, Sun W, Qian W, Ye Y, Ma X (2009). The synthesis of chitosan-based silver nanoparticles and their antibacterial activity. Carbohydr Res.

[25] Lopez-Flores AI, Velazquez-Enriquez U, Scougall-Vilchis RJ (2025). A new disinfection approach using a chitosan-based endodontic irrigant. Materials (Basel).

[26] Celikten B, Amasya G, Oncu A, Koohnavard M, Saklar F (2022). Effects of chitosan-containing silver nanoparticles or chlorhexidine as the final irrigant on the bond strength of resin-based root canal sealers. J Dent Res Dent Clin Dent Prospects.

[27] Ratih DN, Sari NI, Santosa P, Kaswati NM (2020). Time-dependent effect of chitosan nanoparticles as final irrigation on the apical sealing ability and push-out bond strength of root canal obturation. Int J Dent.

[28] Carrilho MR, Carvalho RM, Sousa EN (2010). Substantivity of chlorhexidine to human dentin. Dent Mater.

[29] Ivanova N, Ermenlieva N, Simeonova L, Kolev I, Slavov I, Karashanova D, Andonova V (2023). Chlorhexidine-silver nanoparticle conjugation leading to antimicrobial synergism but enhanced cytotoxicity. Pharmaceutics.

[30] Lu MM, Wang QJ, Chang ZM (2017). Synergistic bactericidal activity of chlorhexidine-loaded, silver-decorated mesoporous silica nanoparticles. Int J Nanomedicine.

